# Estimation of PSD Shifts for High-Resolution Metrology of Thickness Micro-Changes with Possible Applications in Vessel Walls and Biological Membrane Characterization

**DOI:** 10.3390/s121115394

**Published:** 2012-11-09

**Authors:** Antonio Ramos, Ivonne Bazán, Carlos Negreira, Javier Brum, Tomás Gómez, Héctor Calás, Abelardo Ruiz, José Manuel de la Rosa

**Affiliations:** 1 Laboratorio Señales, Sistemas y Tecnologías Ultrasónicas, Consejo Superior de Investigaciones Científicas (CSIC), 28006 Madrid, Spain; E-Mails: tgomez@ia.cetef.csic.es (T.G.); hcalass@gmail.com (H.C.); artuscu@hotmail.com (A.R.); 2 ESIME, Instituto Politécnico Nacional (IPN), 07738 México DF, Mexico; E-Mails: trujilloivonne20@gmail.com (I.B.); mdelaros@ipn.mx (J.M.R.); 3 Departamento de Materiales, Facultad de Ciencias, Universidad de la Republica, 11400 Montevideo, Uruguay; E-Mails: carlosn@fisica.edu.uy (C.N.); jbrum@fisica.edu.uy (J.B.)

**Keywords:** spectral metrology, transducer systems, systems integration, high-resolution, non-invasive estimation, wall and membranes thickness, PSD shifts

## Abstract

Achieving accurate measurements of inflammation levels in tissues or thickness changes in biological membranes (e.g., amniotic sac, parietal pleura) and thin biological walls (e.g., blood vessels) from outside the human body, is a promising research line in the medical area. It would provide a technical basis to study the options for early diagnosis of some serious diseases such as hypertension, atherosclerosis or tuberculosis. Nevertheless, achieving the aim of non-invasive measurement of those scarcely-accessible parameters on patient internal tissues, currently presents many difficulties. The use of high-frequency ultrasonic transducer systems appears to offer a possible solution. Previous studies using conventional ultrasonic imaging have shown this, but the spatial resolution was not sufficient so as to permit a thickness evaluation with clinical significance, which requires an accuracy of a few microns. In this paper a broadband ultrasonic technique, that was recently developed by the authors to address other non-invasive medical detection problems (by integrating a piezoelectric transducer into a spectral measuring system), is extended to our new objective; the aim is its application to the thickness measurement of sub-millimeter membranes or layers made of materials similar to some biological tissues (phantoms). The modeling and design rules of such a transducer system are described, and various methods of estimating overtones location in the power spectral density (PSD) are quantitatively assessed with transducer signals acquired using piezoelectric systems and also generated from a multi-echo model. Their effects on the potential resolution of the proposed thickness measuring tool, and their capability to provide accuracies around the micron are studied in detail. Comparisons are made with typical tools for extracting spatial parameters in laminar samples from echo-waveforms acquired with ultrasonic transducers. Results of this advanced measurement spectral tool are found to improve the performance of typical cross-correlation methods and provide reliable and high-resolution estimations.

## 1. Introduction. Piezoelectric Metrology and Working Hypotheses

Investigation lines to create new applications of piezoelectric electromechanical transducers in both time and frequency domains, seeking accurate measurement of internal physical magnitudes in diverse materials, are the subject of increasing research. This is because of their potential capability for solving numerous metrological problems in diverse scientific, industrial and medical areas. The use of spectral techniques applied to the raw measured data opens up the possibility of increasing the precision in the analysis of the electrical signals provided by this type of piezoelectric devices. Particularly useful applications are the precise metrology of internal distances and of plate thickness inside opaque pieces. Examples in the medical field would be the accurate measure of early inflammation levels in biological tissues, of possible preliminary changes in biological membranes thickness (amniotic sac and parietal pleura) and of thin walls in certain body tissues (for instance, blood vessels [1,2]). In fact, this would provide the technical basis to analyze the possible technological achievement of early diagnostic tools intended for dangerous diseases such as atherosclerosis, hypertension or tuberculosis.

In the field of the membranes research and its applications, both frequency and time techniques (e.g., time domain reflectometry TDR) have been used to determine early membrane fouling that can be detected by effective thickness changes and variations in other membrane properties. The thickness variation can be measured with good resolution, in the case of membrane compaction and TDR [3–7].

Applications for diagnostic purposes of new metrological procedures based on piezoelectric transducer systems, working in the megahertz range, appear to offer possible solutions and thus allow the mentioned precise spatial measurements in tissues. However, reaching this objective in patients requires accurate estimation (in a non-invasive way) of scarcely measurable parameters related to internal tissues, which presents many difficulties with the current technological “state of the art”. For instance the use of commercial echography equipment, for estimating spatial sizes from tissue images of internal organs, has serious limitations as the resolution is typically worse than 0.5 mm in such instruments (even at working frequencies as high as 7.5 MHz). Units working at higher frequencies, such as 20 MHz, are only adequate for analysis of superficial parts (like the skin, or for use in ophthalmology). Besides, their technological complexity is always high since at least a hundred ultrasonic channels are required: 100 piezo-transducers and their related Emission & Reception (E/R) electronic units. This complexity is detailed in the Section 3 where an efficient implementation of the pulsed electronic control stages for broadband ultrasonic array systems is addressed.

Additional studies based on echographic ultrasonic imaging units and also segmentation algorithms [8–12] have provided improved measurements in vessels. Spatial resolutions so obtainable from these imaging units (worse than 200–300 μm, even after advanced image processing), are not still sufficient as to permit accurate wall thickness estimations with a precise application for early diagnosis in arteries, where variations, so light as 35 μm [11] or 10 μm [12], have a clear clinical interest.

Therefore, specific techniques must be created, not necessarily involving significantly more complexity in the transducers and electronics technology. In fact, they could be based on only one ultrasonic channel (of a topology similar to only one of the “n” channels shown in the Section 3), but specifically intended for one-side echo-estimation into thin layers, membranes and laminated pieces. This can be achieved by adding sophisticated signal processing stages, which are focused on improving the accuracy in spatial measurements ranging around a few microns.

This new option should be applicable to facilitate a non-invasive early diagnosis objective, and also in the more general metrological field of thickness gauging [13,14] in opaque pieces with limited access from only one side. Here, improvements in the conventional ultrasonic one-side piezoelectric meters, to reach accuracies near one micron, would be of a high utility.

Certain similar high-resolution applications of mono-channel and array ultrasonic transducer systems in other medical areas have been preliminarily explored (some of them, very recently) to make possible internal measurements and non-invasive diagnosis of some human diseases [15–24]. They include the case of only one transducer actuating as an ultrasonic emitter and also performing echo detection. A two-way procedure is used with the radiation controlled by a pulsed high-voltage electronic driving [25], detection-matching-tuning circuits and other analogue electronics (decoupling, broadband amplifying and filtering circuits) [26]. The detected spectral echo-information is then processed using custom digital signal techniques [27]. In each new application, an efficient integration of the selected transducers with the interface and transceiver electronic sub-systems is required.

The entire piezoelectric transduction system, so composed, has been shown to be an efficient tool to extract information about tissues in a non-invasive way to achieve different new medical objectives: (i) to provide data for the diagnosis of some viral or degenerative diseases like cirrhosis [15–18]; (ii) to estimate thermal distributions in tissues under hyperthermia cancer treatments by means of an analysis of the time echo-waveforms [19,21–23]. In the last case, the frequency spectral patterns can be successful processed [20,24], with promising further extensions, maybe making possible the detection of some tumors (such as in breast) in their early phases. Thus, neo-vascularizations could be detected from their related light thermal effects, due to increases of blood perfusion in the pre-tumor zone [27].

The *spectral analysis* of ultrasonic echoes can be a low-cost tool to estimate small changes of anatomical and physiological parameters modifying spectral peaks, due to the interactions of emitted ultrasonic pulses with the granular, dispersive (or as in our case, laminar), nature of certain tissues.

These ultrasonic echo-pulses present rather complex time and frequency distributions making, a direct interpretation of the related tissue information, difficult, but just the spectral estimation is a general purpose tool employed to analyze with improved resolution complex or noisy radiofrequency time-signals. In fact, it has been probed as an effective option to obtain data hidden inside noisy echoes related to tissue structures, for instance in non-invasive thermometry [24,28].

Some research lines in spectral ultrasonic metrology are oriented at determining the average distance among scatterers, using high-resolution PSD (power spectral density) estimation [29]. Other current research attempts to associate overtones frequency behavior with temperature changes in a tissue [20].

In applying this processing tool to noisy bio-ultrasonic signals, some problems (derived from the rather complex nature of the acquired signals) can be analyzed. For instance, the echoes are not always of a clearly deterministic and totally regular type. In tissues, the required information could be mixed with noise and undesirable masking perturbations, so accuracy in measuring peak frequencies in echo spectra could be affected by unfavorable elements creating spurious frequency peaks on PSD.

In order to properly evaluate here these aspects and some advantages of this type of tools, several previously reported analysis procedures [24,28] will be extended to this problem. These procedures were intended for thermal measurement at the internal parts of tissues, or phantoms designed with internal multiple scattering that emulates the texture of some human organs.

Our *starting hypothesis*, in the current work, is that the changes in the ultrasonic parameters to be evaluated in each application could be obtained by applying the developed spectral techniques. They are similar to those described above for other type of clinical applications, but adapted to the new conditions because of a common circumstance: the tissue changes appear reflected on certain time and frequency variations encountered between normal multi-pulse echo-traces and those acquired from pathologic tissues. Here, these variations would be related to light inflammations in membranes or micro-changes in thickness of vessel walls. The changes, in amplitude and phase of spectral peaks in the echoes, could be caused by: (a) modifications in the tissue stiffness (fibrosis processes), (b) internal distance changes among scatterers in hepatic tissues (as potential indicator of chronic hepatitis), (c) variations in the thickness of artery walls (due to atherosclerosis or atheroma plaque formation).

## 2. General Aspects and Planning of the Article

In this article, broadband ultrasonic procedures (using spectral techniques developed for other medical detection problems) are specifically modified and adapted to analyze their applications for accurate thickness estimation of sub-millimeter membranes, thin layers or walls. This will be studied in phantoms made of materials with acoustic properties similar to those encountered in biological tissues. The technology associated with the experiments involved is based on the integration of a piezoelectric transducer and related electronics into a whole metrological system working in the frequency domain. The main objective of designing the proposed system and procedure is to provide high resolution for the mentioned thickness measurements with mainly medical purposes. It will be explained as follows: along the article sections, the addressed topics are structured to develop three main objectives: (a) a description of the basic pulsed transducer systems to be applied in this work, of their design bases and of aspects of transduction and signal modeling; (b) the application of specific electronic and processing techniques for noninvasive metrology of distances and sizes inside thin tissues (detecting frequency alterations in ultrasonic echo-information); (c) the study of most important factors to be taken into account to establish our improved spectral analysis procedure. The final aim is increasing the basic axial spatial resolution and therefore the resulting precision in thickness measurements. For assessing it as future estimation tool for accurate medical diagnosis in biological membranes and blood vessels, resolutions, accuracy, and robustness aspects (including noisy conditions), will be considered.

Certain advanced processing options to estimate overtones location in power spectral densities are quantitatively evaluated for echo-responses acquired in laminar pieces with a broadband piezoelectric transducer of 10 MHz in nominal frequency, operated under transient electronic driving, and also with calculated echoes computationally generated from reliable multi-echo models.

The main objective of this comparative analysis is to determine the contributions of each option to the resulting potential resolutions of the complete thickness measuring system for opaque specimens, investigating its capability to provide spatial accuracies around 1 micron. The measurement resolutions so attained are compared with those obtained, using the same piezoelectric transducer and similar electronic transceiver hypotheses, but applying conventional signal processing methods in time and frequency domains, for the extraction of spatial parameters from ultrasonic echoes received in laminar samples. Concretely, some estimation results from using conventional cross-correlation techniques (well-known as useful time-delay estimators [23,30,31]), and of applying advanced frequency-domain methods (specifically improved for our purpose [27]) are shown. They provide, in some options of the second group, high-resolution estimations as required for the here selected problems.

In the applications addressed in this work, for accurate thickness metrology, advanced spectral analysis of multi-echo signals is extended to evaluate the frequency peaks' behavior associated to the acoustical resonance induced into the inspected material and including its overtones. These are related with internal spatial parameters of a determined laminar sample, like a biological membrane, a sheet of a new material, or a sanguineous vessel wall. When a change occurs in the physical dimensions of the internal structure of such specimens, certain variations in the resulting ultrasonic echoes (coming from the major internal discontinuities) are also produced: in the echoes times-of-flight or in the density of echoes included into a fixed time period. Then, correlated alterations must also appear in the frequency spectrum of echo-traces obtained as response to a pulsed ultrasonic beam perpendicularly emitted to tissue external surface. This is due to concentrations or expansions in time of the reflected pulses, proportional to the magnitude of the changes to be sensed in the ultrasonic propagation direction [32].

In consequence, these modifications on the time waveforms can be detected as precise changes in the location of overtones related with the fundamental internal resonance in the analyzed medium, which are tied to the internal spatial parameters to be estimated. Thus, these spectral changes must be very finely analyzed to find their relation with internal alterations of some dimension, originated by particular tissue pathologies. It has direct clinic implications, associated for instance to inflammation, vessel wall widening, tissue density changes, or thermal dilatation by blood irrigation variations; but before making a robust application of the extended technique to advanced medical diagnosis (detecting light changes in membranes and walls), some pending research aspects have to be solved and quantitatively evaluated under laboratory conditions. Particular adaptations of our previous estimation techniques to laminar samples must be introduced, and aspects related to frequency resolution of the ultrasonic procedure intended for spectral metrology purposes must be improved.

Along the paper sections, a study is performed about results obtained for thickness estimation, by adaptation of different temporal and spectral techniques (classical and also of high-resolution) to echo-waveforms acquired from a real thin wall phantom, constructed *ad-hoc* with layers of latex material. The objective is to evaluate the new procedure (under conditions similar to those encountered in real tissues), as a future tool in medical diagnosis, estimating dimensions inside tissues of a laminar or membrane type. The dependence of variations in peak frequencies of the echo spectrum with widening or narrowing in the vessel walls, is analyzed with a tube subjected to a pulsatile flow. A-scan registers are obtained for distinct situations under precise control (to simulate pathologic alterations in the real blood vessel walls). Comparative studies are also made showing how the application of our improved spectral procedure, with broadband transducers, provides a good spatial estimation in a dynamically modified wall thickness. Finally, others echoes computationally calculated by means of numerical simulation of multi-pulse waveforms coming from laminar pieces [32] are also processed.

## 3. Systems Based on Pulsed Piezoelectric Transducers for Noninvasive Reliable Metrology

**Figure 1.**
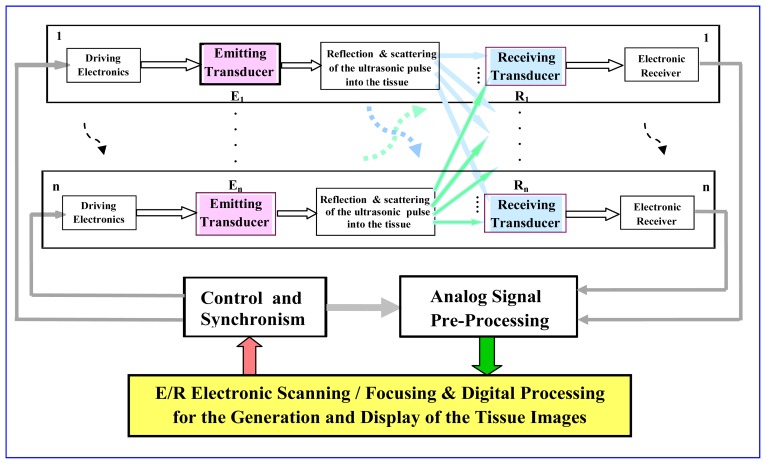
Schematic diagram of the systems developed for ultrasonic echo-imaging using arrays of *n* transducers.

Scanning ultrasonic methods based on the combination of multiple piezoelectric transducer systems (as it is depicted in the block diagram of Figure 1) have been largely investigated for non-invasive exploration of the human body, giving images with good spatial resolutions. For this, they use: (a) fast electronic Scanning (Steering or Mux-DMux) and Focusing procedures for an adequate and precise ultrasonic beam forming [33,34], and (b) additional techniques for imaging construction and its enhancement similar to those used in other medical fields.

The diverse implementations of these rather complex ultrasonic methods have made possible a very ample commercial implementation of conventional echography equipment in hospitals for general purpose diagnosis applications. They are based on displaying, at different colors or gray levels, distinct echo-amplitudes associated to density changes detected by the ultrasonic beam during its propagation through a human organ or a particular tissue. In addition to classical imaging technologies, some proposals were reported to provide more specific ultrasonic tools for certain tissue characterizations or facilitate complementary diagnosis of human diseases [16,17,19–23,35]. They use a direct processing of the pulsed ultrasonic waveforms acquired from the analyzed medium, but searching other different tissue physical parameters such as elasticity [15,16,18,35] or temperature [19–23], being more sensible than the tissue densities with regards to some pathologic states.

By using this last type of characterization tools, complementary and useful information about biological tissues can be easily extracted, which could make possible (in the future) more precise diagnosis methods of viral or degenerative diseases; for example by a non-invasive estimation of elastic modules or of thermal distribution into tissues from time and/or spatial changes in echoes.

These current research lines would be in conditions of defining solutions for an early detection of some types of tumors or degenerative lesions in the near future. In these cases, the parameters being analyzed before performing the display of the measurement results can be (instead of the classical echo amplitude) the ultrasonic speed, the times-of-flight between echoes, the complex amplitude or phase of the echo-spectra, changes of material stiffness or of distances between internal scatterers, *etc*.

### 3.1. Transducer Systems Topology for Detection and Measurements of Ultrasonic Echo-Signals

**Figure 2.**
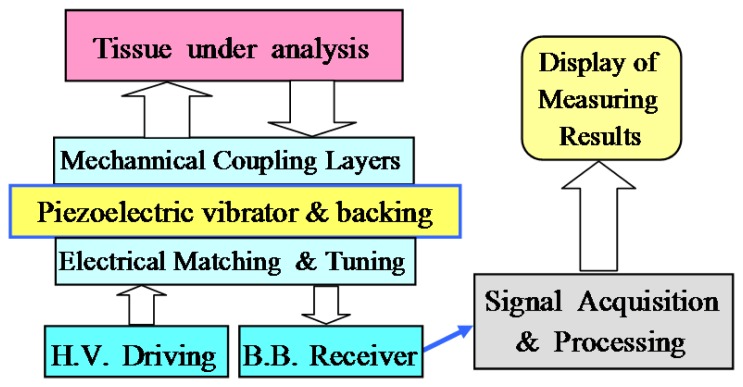
Blocks of a monochannel measurement ultrasonic system using piezoelectric transduction.

The general topology of a basic mono-channel ultrasonic system, using a bidirectional piezoelectric transduction for measuring internal characteristics into biological tissues, is depicted in Figure 2. In this type of applications, the piezoelectric transducers must to be specifically adapted to:

- the acoustical impedance of the radiated medium (which ranges around 1.5–1.6 × 10^6^ Kg m^−1^ s^−2^, in soft tissues), using λ/4 coupling layers of impedances properly chosen for interfacing with tissues.
- the electrical impedance of emitting and receiving electronics, by means of adding electrical matching and tuning networks for interfacing with the high-voltage (HV) driving (supplied from special pulse generators) and with the broadband receiving electronic subsystem.

Some tissues introduce high acoustic attenuation for frequency windows located into the rather narrow working bands of the internal piezoelectric transducer vibrators. Besides, there is other important reason imposing the broadband condition on the detection transducers: the ultrasonic responses have to be of very short duration, to be capable of discriminating internal reflectors located in close positions.

Thus, the involved ultrasonic device must to be of broadband type, which is usually attained by introducing acoustical losses in the piezoelectric vibrators. As a direct consequence, the broadband piezoelectric transducers employed for biological measuring applications must have:

- high efficiency, good signal-to-noise ratio (SNR), and a driving with high-voltage electrical pulses [36];
- a multilayer internal structure, due to the original rather narrow frequency bands, in the piezoelectric ceramics, must be drastically increased by sticking: (i) one or two mechanical coupling layers in the ceramic frontal face [37] as it is shown in Figure 2, and (ii) an acoustically absorbing backing material in the rear ceramic face [38].

All these desired aspects, for the ultrasonic transducers responses, are function of the characteristics of the piezoelectric vibrator and its front and back mechanical layers contained in the transducer; but also strongly depend on the parameters in the electronic generator of the HV driving pulses (pulser), the electrical matching and tuning networks, and the input impedance in reception electronics. Some of these circuits can be of non-linear character, which complicates the analysis and design of the transducer systems, but by a proper choice of electronic parameters and a specific design of transducers for each application, an optimization could be achieved in amplitude and bandwidth of the measurement echo-signals [33,36]. These relevant improvements in both parameters are attained by numerical prediction of an optimal setting at least for the reactive components in the tuning and matching of Figure 2.

### 3.2. Modelling a Real Pulse-Echo Piezoelectric Transducer System for our Metrological Design

There are complex dependences among all the electrical and mechanical parameters of transducers and electronic stages needed for non-invasive metrology [25]. For this, it becomes necessary to use accurate models for the E/R transduction processes and for the interfacing electronics during the design of the whole transducer system. Classical modeling approaches supposed ideal electrical driving with linear and resistive behavior in the pulse generator and parallel loading impedances, but practical ultrasonic units for tissue inspection require more complex global models including: (a) the real non-linear high-voltage excitation circuits and matching and tuning elements included in the electrical blocks of Figure 2; (b) some non-ideal electrical impedances [39]; and (c) accurate models to properly represent the real ultrasonic transducers responses, even accounting for quadratic frequency dependences of the effective mechanical losses into the piezoelectric vibrators and also of propagations through some inspected solid media (both appearing in many practical inspections).

**Figure 3.**
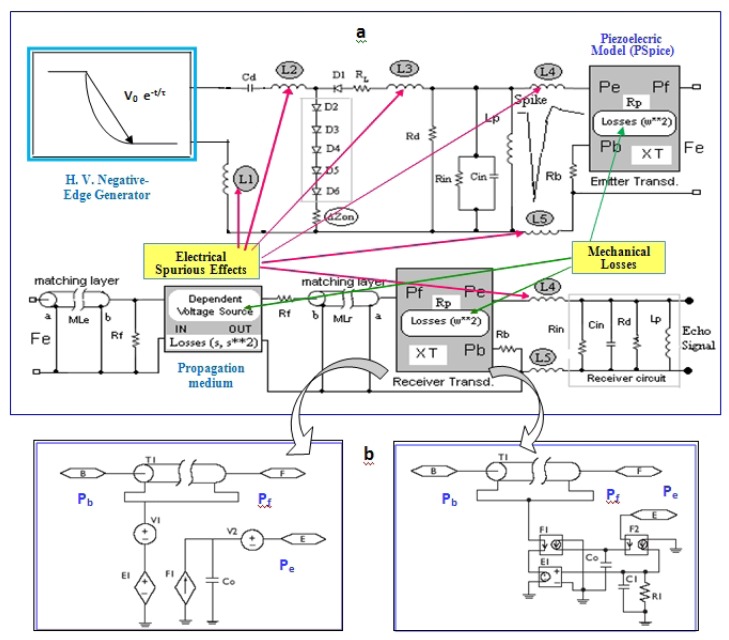
(**a**) Global equivalent circuit modeling for two-way piezoelectric transducer systems, including mechanical losses and ultrasonic & electronic spurious effects. (**b**) Two PSpice implementations of the Redwood model for piezoelectric stages.

A global model for achieving an accurate simulation of the responses expected in transient regime, for the emission/reception stages of pulsed piezoelectric transducer systems was proposed in [40]. It includes all above-mentioned non-ideal aspects [25], and equivalent circuits in P-Spice format for analysing the complete ultrasonic emitter and receiver stages. Quadratic losses in both piezoelectric stages and optionally linear/quadratic losses in the propagation medium are considered. This realistic circuit option for modelling transducer systems (under effective working conditions) is shown in Figure 3(a). The electrical section modelling the pulser subsystem contains (besides the typical matching (R_d_) and tuning (L_p_) components), a HV edge generator (negative ramp/exponential function) with a discharge capacitor C_d_, and other non-conventional aspects (absent in previous simplified transmitter models, but having an important role in transduction behaviour).

This circuital model considers distinct contributions to transducers responses, appearing in the practice when they are connected to real electronic units, and related to:

- Non-ideal impedance elements in the grounding of the circuital branch containing several series semiconductor elements D_i_, under high peaks of current: (ΔZon).
- Parasite inductive impedances from cabling (L_4_ and L_5_), and some critical segments of printed circuit tracks (L_1_-L_3_) in the HV pulser board (which becomes relevant under HF conditions).

In piezoelectric and ultrasonic parts, some models are used: (a) Two transmission lines (MLe and MLr) representing mechanical layers for acoustic impedance matching with the propagation medium (R_f_) in emission and reception. (b) Effects of very simplified diffraction, reflection and attenuation in the inspection medium (in the gain of a dependent voltage source representing external propagation). (c) Two PSpice piezoelectric modelling blocks in Figure 3(a) symbolizing the emitting & receiving resonators with a backing section (R_b_). They use an improved version of the classical PSpice models proposed in [41,42]. F_i_, and E_1_ are dependent sources of current and voltage, and Pb, Pf and Pe are the mechanical (rear section, frontal medium) and electrical ports of the piezoelectric transducer.

The main improvement included in this modeling part is based on considering a quadratic approach for the frequency dependence of the mechanical losses into transducers resonators. This is an option, alternative to the classical linear-dependence implementations [42], which was theoretically derived in [40], after analyzing some experimental measurements in piezo-ceramics reported in [43].

The PSPICE implementation of this quadratic approach allows the introduction of frequency-dependent parameters in the transmission-line with losses T_1_ appearing in the Figure 3(b), which can support the Laplace function. This quadratic-losses approach allows one to calculate echo-responses more in consonance with the really measured ultrasonic signals, by attenuating the influence of the ideal thickness odd-overtones [40], as it really happens in the practical and commercial transducers.

## 4. Simulating Echoes Patterns from Biological Phantoms only for Comparative Analyses

For properly optimizing echo-responses related to a particular metrological design in this area, the modeling guides summarized in Section 3 must be used, but for the rather academic comparative analyses to be made in this work, a simpler, operative and repetitive way to obtain adequate echo patterns is to directly simulate the typical echo-waveforms, in an analytical way from a mathematical expression. Another useful solution, giving repetitive and reliable bio-echo-patterns, is by using the classic phantoms, e.g., of plastic material, in this case emulating arterial walls and thus producing real experimental echoes.

**Figure 4.**
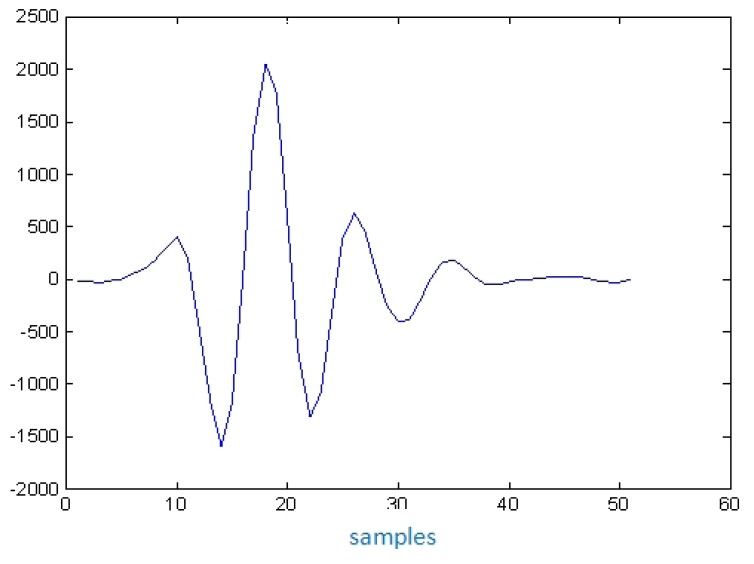
Echo waveform in typical pulsed ultrasonic inspections, measured with a 10 MHz transducer, at the interface between water and a low acoustic attenuation material mimicking an arterial wall.

In the more typical ultrasonic inspection of vessel tissues, the echoes length used are quite short, as can be appreciated in Figure 4, corresponding to an echo-signal measured from the interface of a latex wall immersed in water, in a layered phantom of a blood vessel constructed in our laboratory.

The usual laboratory ultrasonic practice for research in this biomedical field uses distinct types of biological phantoms with internal walls or reflectors approximating tissues under study in what regards their echo-responses. For computer simulation of these responses in propagation media, some authors explain the nature of the ultrasonic signals normally acquired from biological materials, using a model consisting of structures regularly spaced among them [44,45], or well as a collection of randomly distributed scatterers. Others options try to model it as a random distribution of the scattering with certain statistic regularity for signals acquired from tissues with semi-regular structures [46,47].

In a similar way, for the cases studied in this work (layers, walls and membranes), a very simplified modeling (easier to apply than those mentioned in Section 3.2) would be sufficient, only for the effects of our comparative analyses of the proposed thickness measurement technique. Here, the echo-signal waveforms could be considered as a superposition of several (regularly spaced) similar single echoes (with the same or opposite polarities); they would be originated from the interaction among an emitted ultrasonic pulse and its successive reflections by walls or layers in bio-membranes or blood vessels.

A simple and useful modeling, for direct emulation (without needing to use the complex model of Section 3.2) of generic multi-pulse echoes (for reference waveforms), could be obtained by approaching our problem to a set of ideal wall echoes separated by a regular distance; and then, the whole echo-trace would be constructed by a sum of successive and repetitive pulses received from the reflecting walls (each pulse having a different time-of flight, depending on the distance and reverberation number).

For this approach, the emitted unitary pulse can be mathematically modeled as in [47]:

(1) $$e(t)=-te^{-4Bw^{2}t^{2}}\sin(\omega_{0}t)\;t>0$$

where *Bw* is the desired bandwidth and *ω*_0_ is the central angular frequency of the pulse.

Finally, each elemental echo, produced by the interaction of the emitted pulse *e*(t) with a plane reflector, can be considered under certain a approximation (neglecting diffraction effects), as a replica of the emitted unitary pulse, depending its polarity on the respective media impedances.

**Figure 5.**
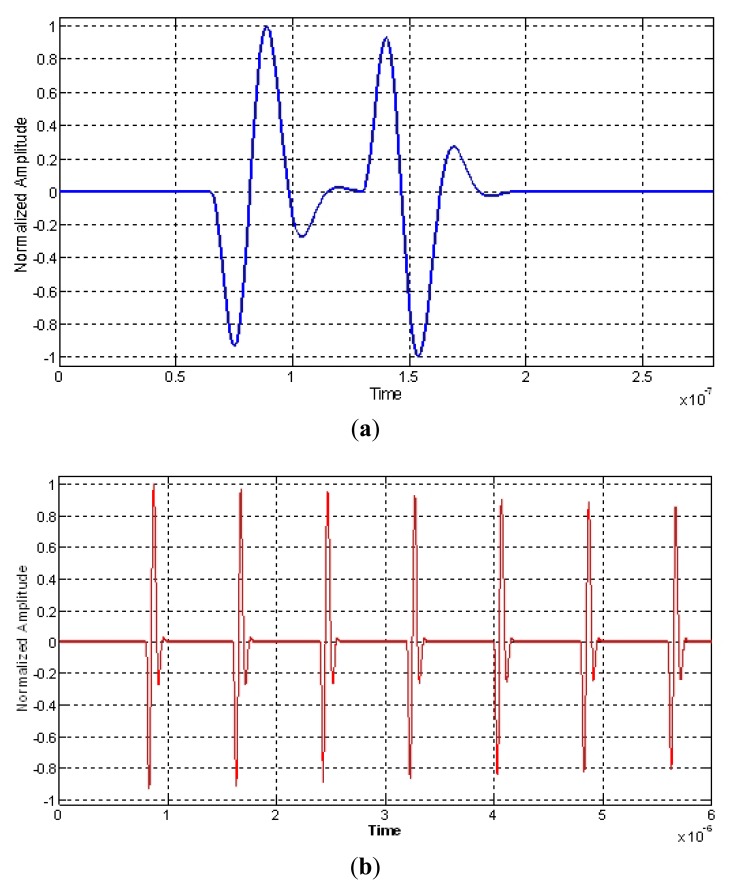
Simulated multi-pulse echoes based on a simple mathematical model of regularly spaced reflectors, emulating: (**a**) two parallel membranes (separated around 50 microns) in an amniotic sac or in a pleura using a 30 MHz pulse; (**b**) uniform reflectors distribution in an ideal tissue (10 MHz pulse).

Two examples of multi-pulse echo-waveforms, simulating ultrasonic signals acquired from different kind of biological tissues, are shown in Figure 5. They are associated to broadband transducers of 30 and 10 MHz in nominal frequencies with 70% in bandwidth; each echo was generated by using the expressions (1). This “simplified” echo simulation is of an ideal type, but it is very useful in the rather academic context of this research work. By using it or alternatively phantom echoes, it can be made under a rigorous control, comparisons among the behaviors of alternative transducer systems with distinct design parameters (before doing their final development), or between distinct signal processing methods.

**Figure 6.**
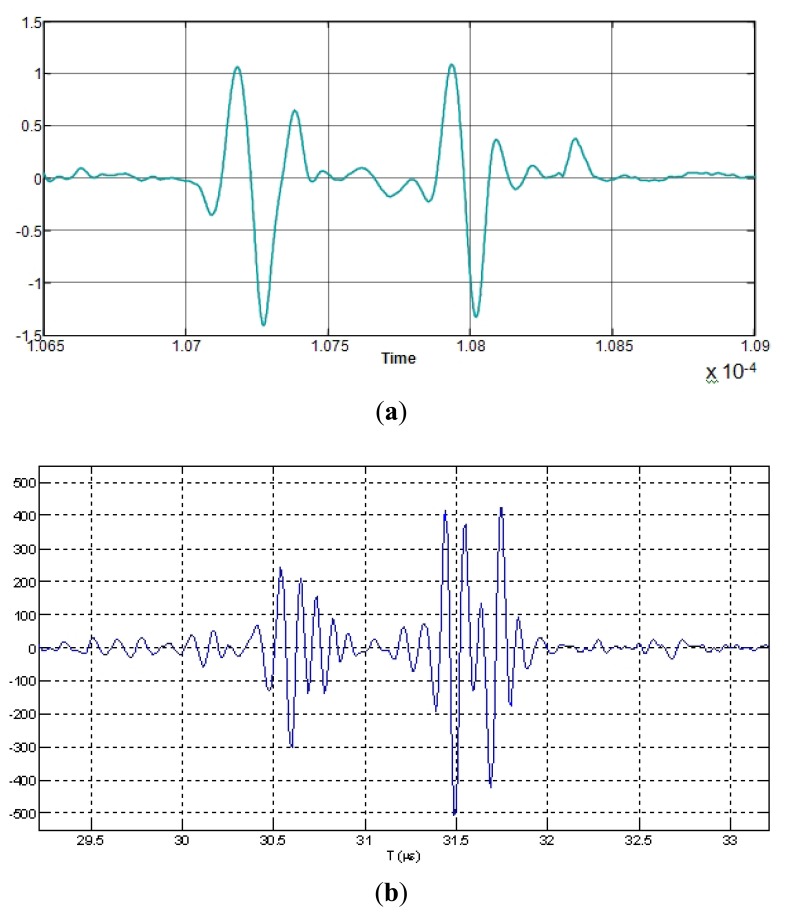
Examples of echo-signals measured with broadband ultrasonic transducers of central frequency f_0_ = 10 MHz: (**a**) from a phantom made with two layers of latex; (**b**) from a femoral artery wall.

Other more realistic reference patterns to study distinct processing methods are waveforms acquired from our phantom (two walls of latex with a fluid inside) or from an artery, as shown in Figure 6. They can be used as a basis for easily introducing (at time or frequency domains) effects of different tissue pathologic alterations in the reference echo-signals for this type of analyses.

Finally, in those cases where a more accurate consideration of the unperturbed exact waveform, related to the used transducers, was needed, then this requirement could be attained by considering the model previously described in Section 3.2 for the distinct stages of a transducer system.

## 5. Applying Spectral Analysis of Ultrasonic Echoes in Measuring Membrane and Wall Thickness

By emitting short ultrasonic pulses into thin walls or layers, with an emitter/receiver system (based on a broadband multilayer transducer properly matched to an efficient pulsed electronic transceiver (like that briefly described in Section 3)), it is possible to estimate the thickness of many pieces with good accuracy. This objective is finally achieved by a special digital processing of the echoes obtained in the ultrasonic reception process, after successive reflections on both faces of the inspected specimen.

For this, parametric algorithms previously developed by the authors to perform harmonic spectral evaluations of biological multi-echo waveforms are here adapted and improved. The aim is to achieve the high frequency resolution required for solving the commented necessities of internal spatial measurements in the medical field. The first advance is an improved algorithm permitting us to apply a new auto-regressive technique in overtones to estimate physical properties of interest for the analysis of wall thickness in blood vessels or of the inflammation level in some tissues.

This type of difficult analyses have an increasing interest for a possible early diagnostic of some diseases and for an accurate estimation of the physical parameters required for the calculation of elastic properties in (rather thin) walls or layers. Some data about thickness estimation will be shown, which were obtained by digital processing of echo-traces acquired in our laboratories (from latex phantoms mimicking vessel properties and also by numerical simulation). It confirms the promising performance for our improved spectral technique in blood vessel characterization. This would propitiate a powerful diagnostic tool, e.g., for early detection of cardiac attacks or of aneurism risk, aspects nowadays receiving a growing attention from medical researchers. Results to be shown in this work suggest clear improvements in the resulting resolutions for this type of measures, in comparison with classical options. In particular, time cross-correlation and non-parametric frequency techniques (currently used to estimate the delays existing between pulsed echo-signals) are considered for the comparisons.

There are some research works reporting laboratory ultrasonic experiments for estimation of wall thickness in blood vessels (for instance: carotid and femoral arteries), with the aim of obtaining an early diagnostic of relevant vascular problems [48–50]. They are related to diseases due to arterial hypertension and atherosclerosis, which often create modifications of the physical properties of the large vessels. This research topic becomes very relevant in some medical sectors due to its potential application in future advanced vascular diagnosis; the related work lines generally include ultrasonic measures of the radial displacement in arterial walls in function of the cardiac pulse. By estimating in the laboratory the mechanical displacements of a phantom wall as a function of internal pressure variations (induced from a pulsed liquid flow), its biomechanical behavior can be analyzed.

The measurements and evaluations required to validate (under laboratory conditions) the mentioned researches were made on phantoms constructed for instance with latex tubes; and in some scant cases, human arteries are also preliminarily characterized in this way [30], showing that when an atheroma plaque is present, a quite different Young modulus appears, than in the case of healthy vessels.

Some ultrasonic procedures have been proposed and applied for evaluating layers and sheets thicknesses, with well-established measurements solution working in time domain; nevertheless, more accurate methods are still needed to respond to new challenges in this technological topic.

In the following paper sections, a new measurement procedure is proposed, capable of detecting the thickness of arterial walls or biological membranes, with an improved resolution. It is based on the use of a modified auto-regressive spectral analysis, for estimating spatial aspects from ultrasonic echoes. The procedure is based on improving and extending a technique proposed in [27] for a quite different medical objective (the estimation of thermal changes in biological tissues containing internal scatterers). This last technique was based on detecting changes in the frequency location of overtones related to echoes spectra acquired from tissues having a regular internal structure. Nevertheless, the viability of this possible wall thickness estimations method, based in the above mentioned hypotheses, must be confirmed with raw ultrasonic echoes difficult to analyze directly; and then, its possible implementation can be decided with ultrasonic transducers, electronic and computer sub-systems. The method performance, with well-controlled echoes coming from laminar or layered phantoms, must be compared with delay measuring techniques previously proposed in both time and spectral domains.

## 6. Delay-Time and Spectrum Based Estimators of Ultrasonic Information Coming from Phantoms

Ultrasonic estimation procedures have been largely investigated as non-invasive tools for diagnosis of some diseases as was detailed in Sections 1 and 2. A research line with promising results is the ultrasonic estimation of thermal distribution in phantoms, and in this context (for other medical necessities) proposals of specific methods have been made by extracting data of interest from internal parts in tissues. They apply direct-time and spectral procedures, looking for the detection of possible morphological alterations from their effects on delays induced on echoes patterns. Such are the cases of: (a) applying the conventional time cross-correlation operator [21,23] for delay estimation between two waveforms; and (b) performing parametric spectral analyses, successfully employed for studying broadband multi-pulse signals [20,24]. In the (b) case, the aim is always to extract clinical information hidden among multiple echoes and speckles, by improving the frequency resolution and facilitating the signal interpretation; in fact, the time-patterns of raw ultrasonic echoes are rather of complex morphology and the tissue information contained in them is difficult to be interpreted with accuracy.

The spectral techniques to be applied in the current work are introduced looking for the solution of new detection problems, with echoes in some way not very different than in previous applications and with similar perturbations as the induced noise and speckles. This could difficult a good discernment among the searched information (of spatial type, in the current case) and undesirable contaminations masking the echoes arrival times (magnitudes to be measured). Thus, an evaluation of reliability for this processing tool would be required before a proper application in a medical diagnostic context.

In consequence, for our particular analyses about thickness estimations, distinct time and frequency domain procedures will be employed to compare their respective responses for improving the current precision levels of conventional ultrasonic methods [13,14], which range around 5–10 microns.

For the time domain case, the cross-correlation operator was chosen as the best option to compare echo-signals for distinct thicknesses in latex phantoms emulating different phases of the cardiac pulse in arteries; this technique appears as the tool to measure delays with better results [30,51]. Though this option is quite robust against signal deformation and moderate SNR levels, any time-domain method has an inherent limitation in resolution for estimation of small delays, related to the sampling period.

A second option for thickness estimation will be tested here, using frequency-domain analyses (of complex echo-signals) proposed for detecting changes on other physiological parameters, like texture and temperature in tissues. They use to be directly related with variations in echo-delays [20,24,52]; in fact, these delays can be quantified by detecting the locations of certain peaks appearing in the frequency spectra. This alternative procedure appears as adequate for delay measure with echoes as those involved here, if conventional basic frequency resolutions in spectral analysis were improved.

### 6.1. Signal Processing Estimators and Operators to be Applied for Thickness Measurement Techniques

In this sub-section, some selected analysis tools implemented in time and frequency domains, for achieving accurate estimations of thickness variations, are proposed and briefly summarized. For obtaining in the *Time domain* a good analytical estimation of the delay between two ultrasonic waveforms (*x, y*) similar in shape but with distinct time occurrences, as those obtained by reflection in the two interfaces of a wall, a classical method based on making a Cross-correlation of both functions can be applied. The true cross-correlation between two discrete signals can be defined in this way:

(2) $${\text{CC}}_{\text{xy}}(m)=E[x_{n}.y_{n-m}^{*}]$$

where *E*[.] is the “expected value”, *x_n_*, *y_n_* are stationary sequences, and *m* is the lag between them.

The delay evolution can be calculated dynamically from the shifts produced in the signals, related to a possible continuous change in the wall thickness. A measure of the delay between *x* and *y* is the displacement existing between the maxima of *CCxy* and *CCxx*, quantified as number of samples.

The main limitation of the thickness estimations based on this operator (though they are intended for advanced delay detection) is the resolution employed to acquire the high-frequency ultrasonic echoes. This reduces the final resolution for the searched diagnosis parameter (in our case, the wall thickness in blood vessels, or tissue inflammations for early detection of infections).

An optional alternative to using cross-correlation is by estimating Shifts into the Power Spectrum (in *Frequency domain*), more concretely their variations with echo time-shifting. In the discrete case, the power spectral density of *x_n_* is related to the autocorrelation (*CC_xx_*) by the discrete Fourier transform:

(3) $${\text{PSD}}_{\text{xx}}(\omega )=\frac{1}{2\pi }\sum_{-\infty }^{\infty }\left[{\text{CC}}_{\text{xx}}(m)e^{-j\omega m}\right]$$

where *ω* = *2πf / f_s_*, being *f_s_* the sampling frequency.

A quite simple spectral estimation method of non-parametric type, known as “Periodogram”, is often employed for finding the power spectrum density (PSD):

(4) $$\text{Prd}(e^{j\omega })=\frac{1}{2\pi N}{\left\{\sum_{1}^{N}x_{n}\;e^{-j\omega n}\right\}}^{2}$$

In the present work, this method will be used to easily measure frequency shifts in overtones related to time-delays between two pulsed signals contained in the same trace (echoes belonging to the two spatial interfaces); but this option shows some limitations in resolution derived from the implied FFT algorithm. For overcoming this resolution constraint, other spectral analyses, but already of parametric types will be here used. The main advantage of using parametric methods for PSD estimation is the achieving of a better frequency resolution without introducing distortion effects due to the windowing.

In order to perform “Parametric analyses”, some auto-regressive (AR) models can be assumed:

(a) One *first approach* for PSD estimation of the AR type (with high-resolution) is based on the Yule-Walker method, which uses the autocorrelation estimates matrix to find the AR parameters [53,54], extrapolating signal autocorrelation values for displacements greater than the signal length. As it is necessary to know “*a priori*” information about how the signal data are generated, a model must be constructed for data generation having certain parameters estimated from the signal data.

In this first parametric method, the data sequence is modeled as the output of a discrete linear system with a transfer function defined at the z domain in the following way:

(5) $$\text{Data}(z)=\frac{1}{A(z)}=\frac{1}{1+\sum_{i=1}^{p}z^{-i}a_{i}}$$

The output of this system is an autoregressive process of order *p*. The model parameters (a_i_) are calculated with the linear Yule-Walker equations. The signal PSD is obtained by the expression:

(6) $${\text{PSD}}_{\text{xx}}(f)=\frac{\sigma_{w}^{2}}{{\left|A(f)\right|}^{2}}$$

where $\sigma_{w}^{2}$ is the input sequence variance, assumed as white noise of average power similar to the unit.

Results obtained using a variant of this AR parametric method, focused on increasing resolution in other distinct non-invasive procedure also based on ultrasonic estimation, have been presented in [24].

(b) A *second approach* for AR-PSD estimation of small frequency changes, improving the previous high-resolution spectral technique, is used here, by adding an ulterior processing step in the acquired waveforms and applying the Burg method, which produces better resolution for our spectral estimation that the other classic spectral approaches. During the estimation of AR parameters by this last method, a minimization is made (based on least squares criteria) of direct and inverse errors in linear predictors. These errors, *d*(n) and *i*(n), for a discrete function *x(n)*, are defined as:
  (7) $$\begin{array}{c} d(n)=x(n)-\hat{x}(n)\\ i(n)=x(n-m)-\hat{x}(n-m) \end{array}$$
  where *x̂*(*n*) and *x̂*(*n* − *m*) are the estimate of direct and inverse linear prediction.

The minimum square error, $\varepsilon_{m}=\sum_{n=m}^{N-1}\left[{\left|d_{m}(n)\right|}^{2}+{\left|i_{m}(n)\right|}^{2}\right]$, can be minimized by selecting the prediction coefficient according to the restriction fulfilling Levinson-Durbin recursion [32]. In this Burg method, the reflection coefficients of the equivalent lattice structure is computed, and the Levinson-Durbin algorithm is used for obtaining the AR model parameters. Finally, based on the AR parameters, the Power Spectrum can be estimated as:

(8) $$P_{\text{xx}}^{\text{Burg}}(f)=\frac{{\hat{E}}_{p}}{{\left|1+\sum_{k=1}^{p}{\hat{a}}_{p}(k)e^{-j2\pi fk}\right|}^{2}}$$

where *Ê_p_* is an estimate of the driving noise variance, *â_p_*(*k*) are AR parameter estimates, and *p* is the model order.

There are three interesting advantages of using the Burg method for estimating the AR model parameters: (i) a higher frequency resolution, (ii) a stable AR model, and (iii) a better computational efficiency.

Our solution to achieve high-resolution in the proposed thickness estimation problem, is completed by adapting to the processed waveform registers (that involves to extract rather short-time windows from the acquired echo-signals) a procedure similar to the employed in techniques of other processing areas. It consist in decomposing each total echo-trace in small fractional time-windows related to each wall or membrane to be analyzed, and then extending their lengths (before and after of the occurrence of each segmental window); null-value samples are added in number enough to attain a signal of *Nw* digital samples, from the original register with *N_i_* samples (*Nw* = *xNi*). For implementing it, each new extended register (*ER_j_*) is arranged by properly modifying the original register, *OR_j_(n)*, in this way:

(9) $$ER_{j}=[0_{1},0_{2},\ldots 0_{(x-1)Ni/2},OR_{j}(1),OR_{j}(2),\ldots OR_{j}(Ni),{0^{\prime }}_{1},{0^{\prime }}_{2},\ldots {0^{\prime }}_{(x-1)Ni/2}]$$

**Figure 7.**
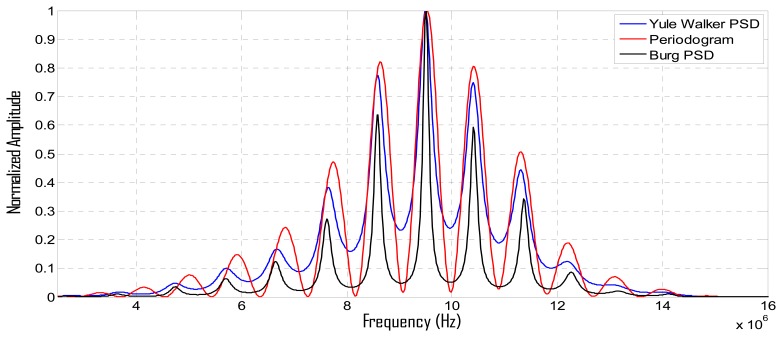
PSD estimations by applying a non parametric technique (Periodogram) and our two parametric options based on the Yule Walker and Burg methods.

PSD estimations, calculated with the above described parametric and non-parametric methods for a two-echoes signal related to a broadband piezoelectric transducer centered around 9,5 MHz, are shown in Figure 7, where the respective methods performances can be comparatively analyzed.

It can be appreciated that: (a) the Periodogram option presents the widest frequency peaks among the three PSD methods, producing a rather poor resolution in the peak frequency determination; (b) our method (using the Burg PSD estimates) provides the narrowest frequency peaks, so optimizing the spectral discrimination for overtones locations, and, in consequence, the potential spatial resolution.

### 6.2. Experimental Setup and Echo-Signal Acquisitions for Ultrasonic Estimation of Wall Thickness

**Figure 8.**
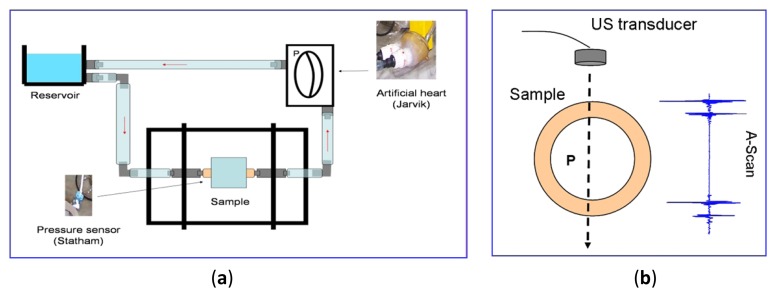
(**a**) Scheme of the experimental set-up for wall displacement measurements (circulating loop mimicking a physiological pulsatile flow inside the arterial phantom). (**b**) A-Scan containing four echoes.

With the aim of calculating in the laboratory the variation in thickness of a latex phantom, emulating the dynamic of the walls in arterial vessels, an experimental setup was constructed, which provides the A-Scan echoes (originated from the wall) needed to estimate wall spatial dimensions. For simulating a cardiac pulse in a controlled way, an elastic latex tube was intercalated in a dynamic circuit (see Figure 8(a)) containing a perfusion line made of polyethylene and silicone connecting the latex phantom to an artificial heart (Jarvik) that generates a variable pressure (from 25 to 125 mm Hg) in the fluid medium.

In addition to the non-invasive thickness measure, elastic parameters in tube walls can be estimated, providing complementary information with a high diagnostic value to assess blood vessel states. For echoes detection during the wall displacements, a broadband ultrasonic probe (Olympus V312SU, 10 MHz in nominal frequency and 70% in bandwidth at 6 dB) was operated in the pulse-eco mode, to generate near-field ultrasonic beams perpendicular to the tube wall, as shown in Figure 8(b).

**Figure 9.**
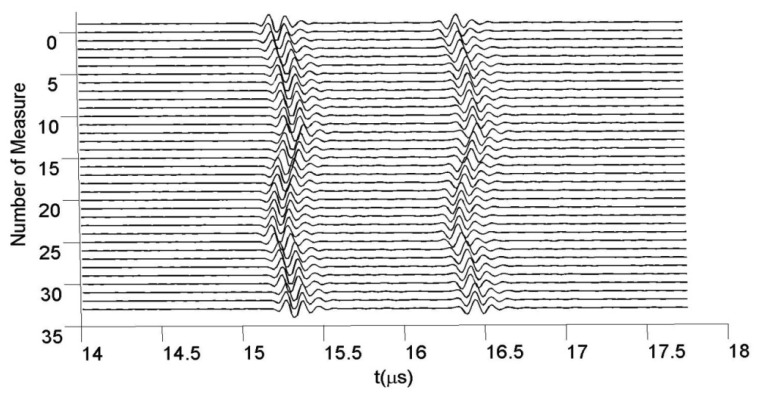
Thirty five time-waveforms belonging to echoes experimentally acquired from the nearest wall to transducer of an elastic latex tube, filled with water under dynamic internal pressures.

For different pressure values, many A-Scans were acquired at 80 MHz in sampling frequency. Figure 9 shows some of the echoes taken from the tube wall zone closer to transducer, for 35 fluid pressures.

The fluid used in our experiments was water, with impedance and velocity similar to blood. Low amplitude speckles from sub-wavelength scattering in blood vessels are not significant in comparison to the clear echo-signals coming from the wall interfaces. In the real vessels walls, micro-reflectors appear, but always it is possible to discriminate the two echoes related to the Intima-Media Thickness (IMT); in fact approximated IMT estimations are currently used in some vessel diagnosis with high-frequency ultrasonic imaging units, but yet with a limited resolution [12].

Measuring the delays between the two echo-pulses in each one of the so acquired A-Scans, by means of a conventional cross-correlation algorithm, the successive displacements, originated in this wall by the externally induced pressure changes, can be approximately calculated in the time domain:

(10) $$\Delta W=v[\Delta t_{0}-\Delta t]$$

where Δ*W* is the wall thickness variation, *v* is the average ultrasound velocity through the medium, Δ*t_0_* = *t_2_* − *t_1_* is the difference detected between times of flight of both echo-pulses in the cycle instant corresponding to the resting tube position (*i.e.*, with the narrowest tube diameter), and Δ*t* is the changing difference between the flight times of both echoes during the tube expansions. For two states of a same vessel, at similar body temperature, possible light changes in the average velocity *v* have a minor influence, and the significant data for diagnosis are the differential changes to be produced.

**Figure 10.**
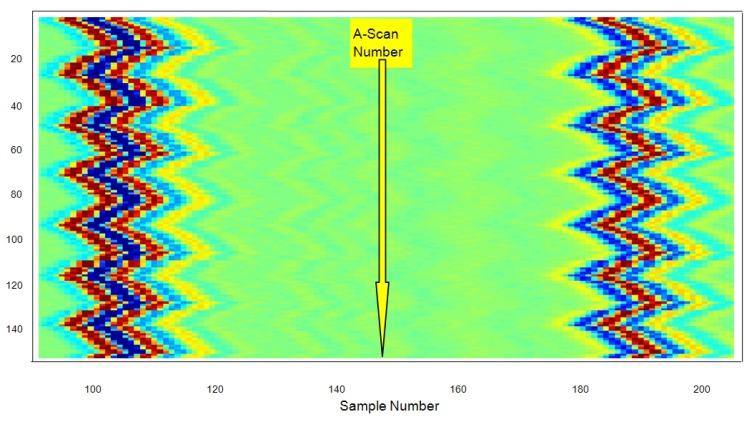
Display in color scale of all 150 echoes from the first tube wall, showing coarse wall changes.

The set of all 150 echoes acquired from the first wall in the latex tube (meanwhile the internal pressure changes) are shown in Figure 10, with a separated line for each A-scan. The approximated value of the resting thickness was ≈0.96 ± 0.01 mm, measured with a conventional caliper. This value was chosen as representative of the carotid artery wall thicknesses, normally ranging between 0.6 and 1.1 mm [55].

While measurements were taken, the internal pressure in the latex tube was periodically varied, to artificially create successive wall displacements similar to those encountered in the real blood vessels.

The effects of the periodic vessel widening on the wall displacements are evident, but possible related contractions in wall thickness are still not visible in this initial graphical analysis of acquired A-Scans.

## 7. Comparison of Results Obtained with Temporal and Spectral Analyses. Some Estimations of Dynamic Thickness and Robustness to Noise for our Approach

**Figure 11.**
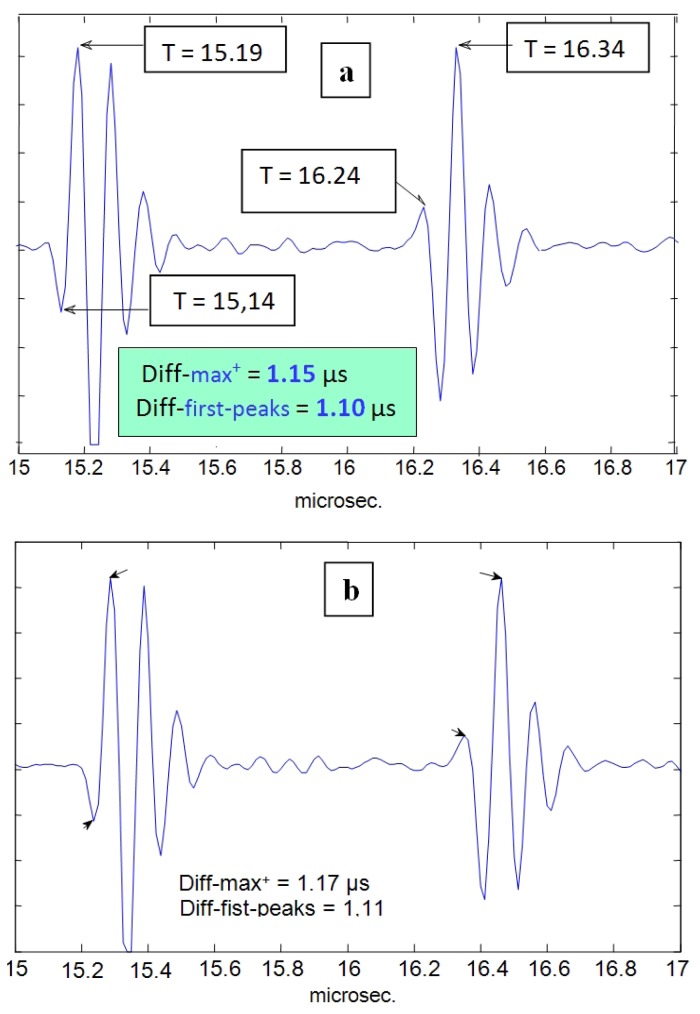
(**a**) A-Scan line number 26 where -maximum tube diameter & minimum wall thickness- occur; (**b**) A-Scan line number 38 where -minimum tube diameter & maximum wall thickness- occur.

In Figure 11(a,b), results of a simple-direct time estimation can be seen (using differences between both maxima or first peaks). With this option, some uncertainty can be appreciated (in the order of 50–60 ns) appearing for the estimation of a precise delay between the two echo-waveforms coming from the two wall interfaces for two distinct cycle instants (maximum and minimum tube diameters).

The time-resolution obtained with this estimation direct-method corresponds (in the spatial domain) to 35–45 microns, on the same order than some results previously reported [56] after performing a sophisticated computer processing of echo-signals obtained from ultrasonic imaging units, where the usual ultrasonic imaging resolution (0.3–0.5 mm) was improved in one order of magnitude. In order to get a further increase of this (already reasonable) spatial resolution, making possible the discrimination of very small changes in wall thickness during the cardiac cycle, additional techniques, more sophisticated than that used to obtain the Figure 11 results, will be applied in the following.

**Figure 12.**
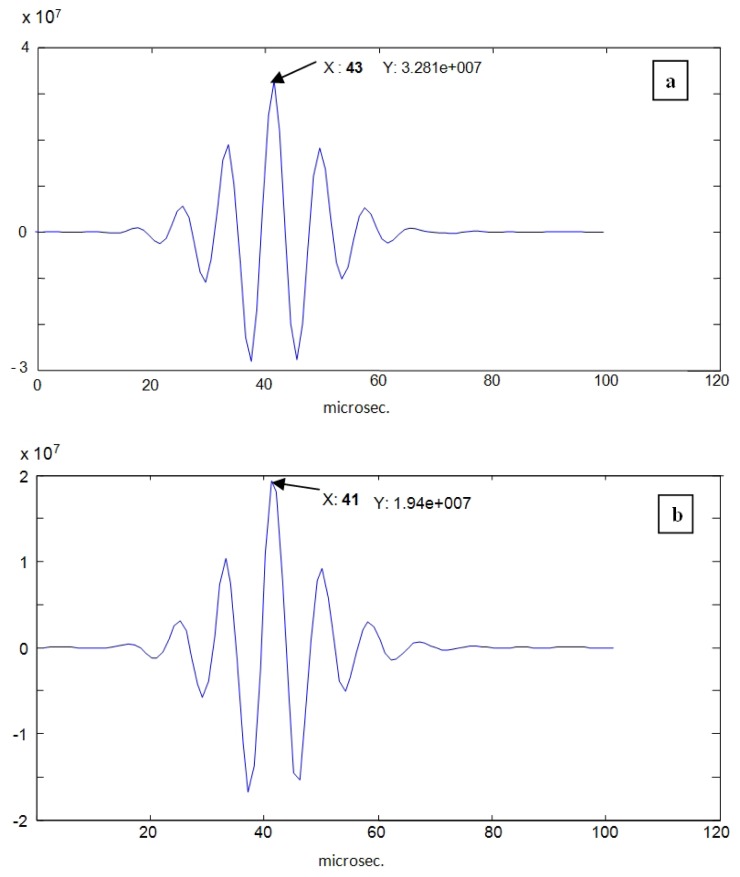
Cross-correlation of echoes in the A-Scans number 26 and 38, for: (**a**) fluid-adventitia interface; and (**b**) intimae-fluid interface.

The first new technique to be applied is defined also in the time domain, and is implemented by means of the classical delay detection method, based on time cross-correlation operations (Equation (2)) between the A-Scan waveforms of the lines 26 and 38 in Figure 10, corresponding to the instants where the narrowing and widening in the tube diameter with major values occur. For these two vessel points analyzed in the Figure 12, which are related to extreme cases into the whole range of variation of the wall thickness, the time shifts for each face (adventitia and intima) of the nearly wall, detected by cross-correlation between the corresponding mono-pulse echoes, were the following:

- In the *adventitia* face of the wall: eight samples (51-43) related to 80 MHz, which is equivalent to 100 ns, measured with a time resolution of 12.5 ns. Thus, the spatial displacement will be: 100/2 ns × 1.8 Km/s = 90 microns, with 12.5% of resolution (equivalent to 11.2 μm).
- In the *intimae* face of the wall: 10 samples (51-41), *i.e.*, 125 ns; and the spatial displacement will be: 125/2 ns ×1.8 Km/s = 112 μm with a resolution of 10% (11.2 μm).

From these data, the applied basic cross-correlation method is already capable of detecting that, at the same time that the tube is being inflated, their walls result compressed in a certain amount; but the value estimated by means of the current method (≈22 μm) still presents an important error of at less ≈ ±11.2 μm, *i.e.*, a percent error of ±51%.

As a first conclusion, this clearly improved resolution (of 11.2 μm) respect to the direct-time method (45 μm), still does not provide sufficiently accurate results to fulfill our initial purpose of analyzing with enough accuracy the changes registered, during a cardiac cycle, on the thickness of the vessel walls under study. On fact, due to this limitation in resolution, some methods based on cross-correlation (for other applications) often must refine their direct results by applying some additional statistical or interpolation techniques [30,31], which performance depends on the level registered for the signal-to-noise ratio of the acquired echoes.

As a second attempt for increasing in a major amount the mentioned spatial resolution, we will apply some methods based on spectral analyses of the echo-responses in the frequency domain. There is a resonance frequency related to the original state of wall thickness, *W_0_*, and the averaged ultrasound velocity on the medium (*v*) defined as:

(11) $$f=\frac{v}{2W_{0}}$$

This reference value in the frequency will be disturbed by any wall thickness variations. The changes in this frequency due to wall thickness modifications are given by:

(12) $$\Delta f=f\left[\frac{-1}{1+\left(\frac{W_{0}}{\Delta W}\right)}\right]$$

where Δ*f* is the frequency change due to a wall thickness variation, *f* is the original resonance frequency, *W_0_* is wall thickness in the original state of the tube and Δ*W* is the wall thickness variation.

In the extended spectral analysis presented in this work, the change in the 10^th^ harmonic peak value will be used for deriving the thickness estimations: Δ*f_10_* = *10* × Δ*f*.

**Figure 13.**
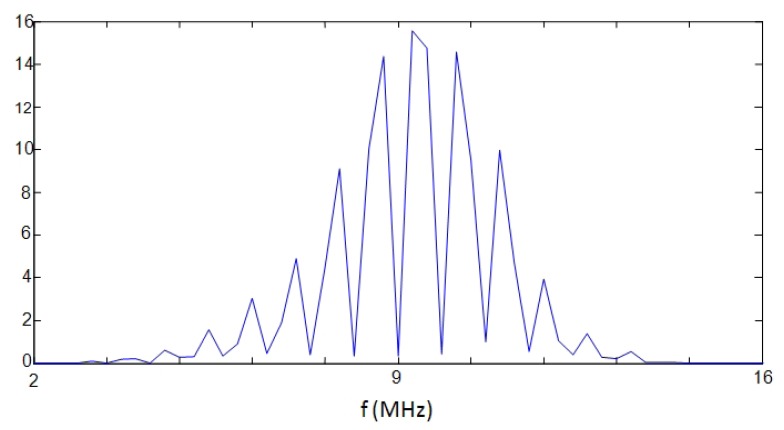
Periodogram of echoes coming from the same tube wall considered in Figures 11 and 12, which is related to the A-Scan number 26.

The more simple options in the frequency domain are based on non-parametric spectral analysis methods, being the Periodogram option one of the most used. As a typical example, a result obtained for the A-Scan line n° 26, applying this spectral option, is depicted in Figure 13. It can be seen as the main peak in the spectral curve results truncated, and 310 kHz of the spectrum are lost, giving a spatial resolution of 405 μm for the fundamental resonance location. This can be substantially improved by analyzing the 10th overtone, which ranges around the center of the transducer band; nevertheless even in this case, the final resolution would be of 40 μm, quite worse than with the correlation method. For this reason, more sophisticated spectral techniques (of the parametric kind) were analyzed looking forward finally achieving the required resolution for our specific problem of spatial estimation.

**Figure 14.**
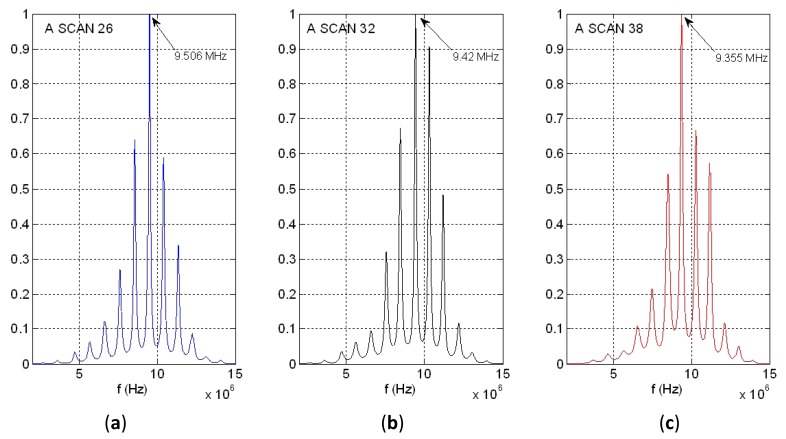
PSD of the tube wall echo related to the A-Scans number 26, 32 and 38 using our method.

Figure 14(a) shows the power spectral density of the same time waveform as in Figures 11–13 (A-Scan number 26), but employing our new spectral procedure. This includes: (a) a time extension of the two-pulse echo received from the tube wall, by applying the expression (9); (b) an autoregressive parametric spectral technique based on the Burg method and using a relatively elevated sampling rate.

### 7.1. Spectral Estimations of a Dynamic Thickness

Resulting peaks in spectrum overtones calculated with our parametric procedure are clearly narrower than with calculations based on the “Periodogram”. By analyzing the 10th overtone in Figure 14(a), like was done for the other spectral option of Figure 13 (with a frequency resolution of 19.5 kHz), a wall thickness of 0.947 mm was estimated, with the achieving of a spatial resolution of (±0.9 μm). This dramatically improves that obtained with the cross-correlation based estimation method (±11.2 μm). A first indication of the procedure repeatability was obtained making other two estimations performed for A-scans 32 and 38, at instants of intermediate and maximum wall thickness in the cyclical tube expansions. The results showing the frequency shifts at the 10th overtone are depicted in Figure 14(b,c). Then, the maximum excursion in the dynamic thickness of our latex phantom was estimated as of 15 ± 0.9 μm.

### 7.2. Robustness to Noise of Our Approach

The ultrasonic estimation of distances inside biological materials, using our approach, is based on the resulting resolution and accuracy for the frequency location in echo overtones of the fundamental resonance. Thus, some alterations in results could appear for not so ideal cases as in Figure 14, e.g., for real tissues, higher frequency pulses and thinner walls. In order to perform a first estimation of the procedure robustness under these unfavorable conditions, this problem was preliminarily analyzed by calculating distinct PSD distributions, for simulated echoes with different levels of added corrupting noise (SNR ranging from 40 dB to 3 dB), a wall thickness of 100 μm, and a broadband transducer of 70% in bandwidth around 30 MHz. The results of these analyses show that for SNR higher that 3 dB, the noise influence in the thickness estimations could be neglected, as it is summarized in Table 1.

As future perspective, if a finer frequency step were used, perhaps spatial resolutions could be achieved improving the found value of 0.9 μm for our approach, even up to one order of magnitude.

## 8. Conclusions

The proposed autoregressive parametric spectral procedure and related ultrasonic system were applied in the laboratory to estimate shifts in PSD overtones of echo-waveforms acquired in a latex tube with a 10 MHz PZ-transducer. Guides were given for technological implementations capable of achieving high-resolution estimation of micro-changes in vessel walls, layers or biological membranes. This presents a growing interest as a complementary tool for future early prevention of cardiovascular accidents.

The application viability of an integrated transducer system, designed for this spectral procedure, was confirmed to detect with accuracy wall thickness changes in a vessels phantom. By improving a parametric algorithm developed by the authors, increasing its sampling frequency for thickness measures, a very good spatial resolution was attained. This was applied to evaluate changes in the phantom, clearly better than with non-parametric spectral techniques. It may be applied for non-invasive calculation of elastic properties in vessel walls, improving the precision currently obtainable with conventional cross-correlation techniques. Models and design rules of transducer systems for this purpose were summarized.

The overtone curves provided by our modified parametric spectral method are clearly narrower than those calculated with the “Periodogram option”. For instance, by using the 10th overtone with a reasonable frequency definition of 19.5 kHz, a spatial resolution of (±0.9 μm) was achieved, clearly improving the spatial performance of the methods based on the periodogram or pure cross-correlation operators.

Results calculated from multi-pulse echo-signals received by a wideband transducer (in the 5–10 MHz range) from a laminar opaque phantom, have shown resolutions better than 1 μm. This was performed for a wall lightly modified in thickness (up to 15 μm) by using periodic inner pressure changes (from an initial value around 0.95 mm). By simulating echoes from a 30 MHz broadband transducer (for a wall thickness of 100 μm), with corrupting noises ranging from 40 dB to 3 dB, it was shown that for SNR higher that 3 dB, noise influences in the thickness estimations are not significant. Nevertheless, new efforts and rigorous overtones analyses with ultrasonic echoes acquired from well-controlled sanguineous tissues patterns are needed, to optimize the potential resolution of this thickness estimation proposal. Its possible clinical limitations must be also evaluated. In particular, its applicability in a clinic context and the performance into real tissues (with “*in vivo*” measured signals) must be assessed.

## Figures and Tables

**Table 1.** (**a**) Values obtained by Burg method for 4th and 5th harmonic peaks of PSD (Hz); (**b**) Percentage error between signals with different level of SNR and signal without noise.

	(**a**)				
**Harmonic Number**	**Without Noise**	**SNR = 3 dB**	**SNR = 6 dB**	**SNR = 20 dB**	**SNR = 40 dB**
4	**30,868.100**	30,877.300	30,874.500	30,904.600	30,882.100
5	**39,355.000**	39,410.100	39,336.400	39,459.500	39,424.600
	(**b**)				
		SNR = 3 dB	SNR = 6 dB	SNR = 20 dB	SNR = 40 dB
4		−0.030%	−0.021%	−0.118%	−0.045%
5		−0.140%	0.047%	−0.266%	−0.177%

## References

[1] Pignoli P., Tremoli E., Poli A., Oreste P., Paoletti R. (1986). Intimal Plus Medial Thickness of the Arterial Wall: A Direct Measurement with Ultrasound Imaging. Circulation.

[2] Wendelhag I., Wiklund O., Wikstrand J. (1992). Arterial Wall Thickness in Familial Hyper-Cholesterolemia Ultrasound Measurement of Intima-Media Thickness in the Common Carotid Artery. Arterioscler. Thromb..

[3] Peterson R.A., Greenberg A.R., Bond L.J., Krantz W.B. (1998). Use of Ultrasonic TDR for Real-Time Non-Invasive Measurement of Compressive Strain during Membrane Compaction. Desalination.

[4] Mairal A.P., Greenberg A.R., Krantz W.B., Bond L.J. (1999). Real Time Measurement of Inorganic Fouling of RO Desalination Membranes Using Ultrasonic Time-Domain Reflectometry. J. Membr. Sci..

[5] Reinsch V.E., Greenberg A.R., Kelley S.S., Peterson R., Bond L.J. (2000). A New Technique for the Simultaneous, Real-Time Measurement of Membrane Compaction and Performance during Exposure to High-Pressure Gas. J. Membr. Sci..

[6] Li J., Sanderson R.D., Jacobs E.P. (2002). Non-Invasive Visualization of the Fouling of Microfiltration Membranes by Ultrasonic Time-Domain Reflectometry. J. Membr. Sci..

[7] Gómez T.E. (2003). Air-Coupled Ultrasonic Spectroscopy for the Study of Membrane Filters. J. Membr. Sci..

[8] Savić Ž.N., Soldatović I., Brajović M.D., Pavlović A.M., Mladenović D.R., Škodrić-Trifunović V.D. (2011). Comparison between Carotid Artery Wall Thickness Measured by Multidetector Row Computed Tomography Angiography and Intimae-Media Thickness Measured by Sonography. Sci. World J..

[9] Groot E., Hovingh G.K., Wiegman A., Duriez P., Smit A.J., Fruchart J.C., Kastelein J.J.P. (2004). Measurement of Arterial Wall Thickness as a Surrogate Marker for Atherosclerosis. Circulation.

[10] Zhang F., Murta L.O., Chen J.S., Barker A.J., Mazzaro L., Lanning C., Shandas R. Evaluation of Segmentation Algorithms for Vessel Wall Detection in Echo Particle Image Velocimetry.

[11] Koskinen J., Kähönen M., Viikari J.S., Taittonen L., Laitinen T., Rönnemaa T., Lehtimäki T., Hutri-Kähönen N., Pietikäinen M., Jokinen E., Helenius H., Mattsson N., Raitakari O.T., Juonala M. (2009). Conventional Cardiovascular Risk Factors and Metabolic Syndrome in Predicting Carotid Intima-Media Thickness Progression in Young Adults. The Cardiovascular Risk in Young Finns Study. Circulation.

[12] Bennett P.C., Gill P.S., Silverman S., Blann A.D., Lip G.Y.H. (2010). Ethnic Differences in Common Carotid Intima-Media Thickness, and the Relationship to Cardiovascular Risk Factors and Peripheral Arterial Disease: The Ethnic-Echocardiographic Heart of England Screening Study. Q. J. Med..

[13] Fowler K.A., Elfbaum G.M., Smith K.A., Nelligan T.J. (1997). Theory and Application of Precision Ultrasonic Thickness Gauging. NDTnet.

[14] Hammond P. (1997). On Resolution, Accuracy and Calibration of Digital Ultrasonic Thickness Gauges. NDTnet.

[15] Boozari B., Potthoff A., Mederacke I., Hahn A., Reising A., Rifai K., Wedemeyer H., Kubicka S., Manns M., Gebel M. (2010). Evaluation of a Novel Ultrasound Method for the Detection of Liver Fibrosis. Prospective Comparison with Transient Dynamic Elastography and Histology. J. Hepatol..

[16] Dasarathy S., Dasarathy J., Khiyami A., Joseph R., Lopez R., McCullough A.J. (2009). Validity of Real Time Ultrasound in the Diagnosis of Hepatic Steatosis: A Prospective Study. J. Hepatol..

[17] Goyala N., Jaina N., Rachapallia V., Cochlina D.L., Robinsonb M. (2009). Non-Invasive Evaluation of Liver Cirrhosis Using Ultrasound. Clin. Radiol..

[18] Castéra L., Bail B.L., Roudot-Thoraval F., Bernard P., Foucher J., Merrouche W., Couzigou P., de Le'dinghen V. (2009). Early Detection in Routine Clinical Practice of Cirrhosis and Oesophageal Varices in Chronic Hepatitis C: Comparison of Transient Elastography (FibroScan) with Standard Laboratory Tests and Non-Invasive Scores. J. Hepatol..

[19] Liu D., Ebbini E.S. (2010). Real-Time 2-D Temperature Imaging Using Ultrasound. IEEE Trans. Biomed. Eng..

[20] Seip R., Ebbini E.S. (1995). Noninvasive Estimation of Tissue Temperature Response to Heating Fields Using Diagnostic Ultrasound. IEEE Trans. Biomed. Eng..

[21] Maass-Moreno R., Damianou C.A. (1996). Noninvasive Temperature Estimation in Tissue via Ultrasound Echo-Shifts. Part I. Analytical Model. J. Acoust. Soc. Am..

[22] Seip R., VanBaren P., Cain C.A., Ebbini E.S. (1996). Noninvasive Real-Time Multipoint Temperature Control for Ultrasound Phased Array Treatments. IEEE Trans. Ultrason. Ferroelectr. Freq. Control.

[23] Maass-Moreno R., Damianou C.A., Sanghvi N.T. (1996). Noninvasive Temperature Estimation in Tissue via Ultrasound Echo-Shifts. Part II. *In Vitro* Study. J. Acoust. Soc. Am..

[24] Bazán I., Vazquez M., Ramos A., Vera A., Leija L. (2009). A Performance Analysis of Echographic Ultrasonic Techniques for Non-Invasive Temperature Estimation in Hyperthermia Range Using Phantoms with Scatterers. Ultrasonics.

[25] Ramos A., San Emeterio J.L., Sanz P.T. (2000). Dependence of Pulser Driving Responses on Electrical and Motional Characteristics of NDE Ultrasonic Probes. Ultrasonics.

[26] Ramos A., San Emeterio J.L. (2008). Interface Electronic Systems for Broadband Piezoelectric Ultrasonic Applications. Analysis of Responses by Means of Linear Approaches. Piezoelectric Transducers and Applications.

[27] Bazán I., Ramos A., Calas H., Ramirez A., Pintle R., Gomez T.E., Negreira C., Gallegos F.J., Rosales A.J. (2012). Possible Patient Early Diagnosis by Ultrasonic Noninvasive Estimation of Thermal Gradients into Tissues Based on Spectral Changes Modeling. Comput. Math. Method. Med..

[28] Bazán I., Ramos A., Trujillo L. Quantitative Predictive Analysis of Responses Obtained from a Strictly Noninvasive Procedure based on Parametric PSD Estimation for Measuring Thermal Properties inside Biological Tissues.

[29] Wear K.A., Wagner R.F., Insana F.M., Hall T.J. (1993). Application of Autoregressive Spectral Analysis to Cepstral Estimation of Mean Scatterer Spacing. IEEE Trans. Ultrason. Ferroelectr. Freq. Control.

[30] Brum J., Balay G., Bia D., Benech N., Ramos A., Armentano R., Negreira C. (2010). Improvement of Young Modulus Estimation by Ultrasound Using Static Pressure Step. Phys. Proced..

[31] Walker W.F., Trahey G.E. (1994). A Fundamental Limit on Delay Estimation Using Partially Correlated Speckle Signals. IEEE Trans. Ultrason. Ferroelectr. Freq. Control.

[32] Bazán I., Negreira C., Ramos A., Calas H., Gómez T.E., Ramirez A., de la Rosa J.M., Gallegos F.J. Possible Application of Spectral Analysis Techniques on Ultrasonic Echo-Traces Improved for Studying Changes in Blood Vessel Walls.

[33] Ramos A., San Emeterio J.L. (2008). Ultrasonic Systems for Non-Destructive Testing Using Piezoelectric Transducers. Electrical Responses and Main Schemes. Piezoelectric Transducers and Applications.

[34] Ramos A., Montero F., Sánz P.T., Torregrosa J.M. (1993). A 5 MHz High-Voltage Demultiplexed Ultrasonic Array System for Rapid-Scan Testing of Advanced Materials. Sens. Actuators A.

[35] Cui L., Shao J., Wang J., Bai J., Zhang Y. (2009). Ultrasound Elastography of Ethanol-Induced Hepatic Lesions: *In Vitro* Study. Chin. Med. Sci. J..

[36] Ramos A., San Emeterio J.L., Sanz P.T. (2000). Improvement in Transient Piezoelectric Responses of NDE Transceivers Using Selective Damping and Tuning Networks. IEEE Trans. Ultrason. Ferroelectr. Freq. Control.

[37] San Emeterio J.L., Ramos A., Sanz P.T., Ruiz A. (2002). Evaluation of Impedance Matching Schemes for Pulse-Echo Ultrasonic Piezoelectric Transducers. Ferroelectrics.

[38] San Emeterio J.L., Ramos A. (2008). Models for Piezoelectric Transducers Used in Broadband Ultrasonic Applications. Piezoelectric Transducers and Applications.

[39] Ramos A., Ruiz A., Sanz P.T., San Emeterio J.L. (2002). Some Non-Linear Aspects of the Electronic Stages in Time Domain Modelling of NDE Pulse-Echo Ultrasonic Systems. Ultrasonics.

[40] Ramos A., Ruiz A., San Emeterio J.L., Sanz P.T. (2006). Pspice Circuital Modelling of Ultrasonic Imaging Transceivers Including Frequency-Dependent Losses and Signal Distortions in Electronic Stages. Ultrasonics.

[41] Leach W.M. (1994). Controlled-Source Analogous Circuits and SPICE Models for Piezoelectric Transducers. IEEE Trans. Ultrason. Ferroelectr. Freq. Control.

[42] Püttmer A., Hauptmann A.P., Lucklum R., Krause O., Henning B. (1997). SPICE Model for Lossy Piezoceramic Transducers. IEEE Trans. Ultrason. Ferroelectr. Freq. Control.

[43] Lethiecq M.L., Tran-Huu-Hue L.P., Patat F., Pourcelot L. (1993). Measurements of Losses in Five Piezoelectric Ceramics between 2 and 50 MHz. IEEE Trans. Ultrason. Ferroelectr. Freq. Control.

[44] Wagner R.F., Insana M.F., Brown D.G. (1987). Statistical Properties of Radio-Frequency and Envelope-Detected Signals with Applications to Medical Ultrasound. J. Opt. Soc. Am. A.

[45] Wear K.A., Wagner R.F., Insana F.M., Hall T.J. (1993). Application of Autoregressive Spectral Analysis to Cepstral Estimation of Mean Scatterer Spacing. IEEE Trans. Ultrason. Ferroelectr. Freq. Control.

[46] Landini L., Verrazzani L. (1990). Spectral Characterization of Tissues Microstructure by Ultrasounds: A Stochastic Approach. IEEE Trans. Ultrason. Ferroelectr. Freq. Control.

[47] Shankar P.M. (1995). A Model for Ultrasonic Scattering from Tissues Based on the K Distribution. Phys. Med. Biol..

[48] Warriner R.K., Johnston K.W., Cobbold R.S.C. (2008). A Viscoelastic Model of Arterial Wall Motion in Pulsatile Flow: Implications for Doppler Ultrasound Clutter Assessment. Pysiol. Meas..

[49] Hasegawa H., Kanai H., Hoshimiya N., Chubachi N., Koiwa Y. Measurement of Local Elasticity of Human Carotid Arterial Walls and Its Relationship with Risk Index of Atherosclerosis.

[50] Bia D., Armentano R.L., Zócalo Y., Campos H.P., Fischer E.I.C., Graf S., Saldías M., Silva W., Alvarez I. (2007). Functional Properties of Fresh and Cryopreserved Carotid and Femoral Arteries, and of Venous and Synthetic Grafts: Comparison with Arteries from Normotensive and Hypertensive Patients. Cell Tissue Bank..

[51] Vera A., Leija L., Tellez A., Bazán I., Ramos A. (2010). Suitability of Alternative Methods of Time Delay Measurements for Ultrasonic Noninvasive Temperature Estimation in Oncology Hyperthermia. New Trends in Electrical Engineering, Automatic Control, Computing and Communication Sciences.

[52] Amini A.N., Ebbini E.S., Georgiou T.T. (2005). Noninvasive Estimation of Tissue Temperature via High-Resolution Spectral Analysis Techniques. IEEE Trans. Biomed. Eng..

[53] Bruce E.N. (2001). Biomedical Signal Processing and Signal Modeling.

[54] Bazán I. (2009). Evaluation and Improvement of Ultrasonic Techniques for Non Invasive Estimation of Internal Thermal Distributions on Biologic Phantoms with Scatters. Ph.D. Thesis.

[55] Lorenz M.W., Markus H.S., Bots M.L., Rosvall M., Sitzer M. (2007). Prediction of Clinical Cardiovascular Events with Carotid Intima-Media Thickness: A Systematic Review and Meta-Analysis. Circulation.

[56] Simon A., Gariepy J., Chironi G., Megnien J.L., Levenson J. (2002). Intima-Media Thickness: A New Tool for Diagnosis and Treatment of Cardiovascular Risk. J. Hypertens..

